# Reactive Oxygen Species in Autoimmune Cells: Function, Differentiation, and Metabolism

**DOI:** 10.3389/fimmu.2021.635021

**Published:** 2021-02-25

**Authors:** Weiji Lin, Pan Shen, Yaqin Song, Ying Huang, Shenghao Tu

**Affiliations:** ^1^Institute of Integrated Traditional Chinese and Western Medicine, Tongji Hospital, Tongji Medical College, Huazhong University of Science and Technology, Wuhan, China; ^2^Department of Emergency Medicine, Tongji Hospital, Tongji Medical College, Huazhong University of Science and Technology, Wuhan, China

**Keywords:** reactive oxygen species, autoimmunity, macrophage, T cell, rheumatoid arthritis, metabolism

## Abstract

Accumulated reactive oxygen species (ROS) directly contribute to biomacromolecule damage and influence various inflammatory responses. Reactive oxygen species act as mediator between innate and adaptive immune cells, thereby influencing the antigen-presenting process that results in T cell activation. Evidence from patients with chronic granulomatous disease and mouse models support the function of ROS in preventing abnormal autoimmunity; for example, by supporting maintenance of macrophage efferocytosis and T helper 1/T helper 2 and T helper 17/ regulatory T cell balance. The failure of many anti-oxidation treatments indicates that ROS cannot be considered entirely harmful. Indeed, enhancement of ROS may sometimes be required. In a mouse model of rheumatoid arthritis (RA), absence of NOX2-derived ROS led to higher prevalence and more severe symptoms. In patients with RA, naïve CD4^+^ T cells exhibit inhibited glycolysis and enhanced pentose phosphate pathway (PPP) activity, leading to ROS exhaustion. In this “reductive” state, CD4^+^ T cell immune homeostasis is disrupted, triggering joint destruction, together with oxidative stress in the synovium.

## Introduction

Oxidative stress represents an imbalance between pro- and anti-oxidants, in favor of the former, and has generally been considered as potentially harmful, since it leads to phenomena including DNA damage, protein oxidation, and lipid peroxidation (1). Based on this dogma, items, such as antioxidant skin care products, natural foods, herbal medicine, and even vitamins have been in demand in recent decades. A typical example of the effects of oxidative stress is ROS-related cell aging. Strikingly, a recent study demonstrated that a modified oxidized form of cysteine residues in proteins is not elevated in old (80 weeks) compared with young (16 weeks) mice, providing strong evidence against the theory of oxidative aging, which involves accumulation of indiscriminate oxidation of biological macromolecules. This study found that protein oxidative state is tissue- and age-specific, and can influence various physiological networks. For example, reversible cysteine oxidation modification of hexokinase controls the flux of glycolysis and the PPP, and polymerization and dissociation of some protein complexes are also regulated by redox state. Researchers have proposed that oxidative modification site, rather than oxidative modification level, is the main target of anti-aging (2).

**Figure 1:**
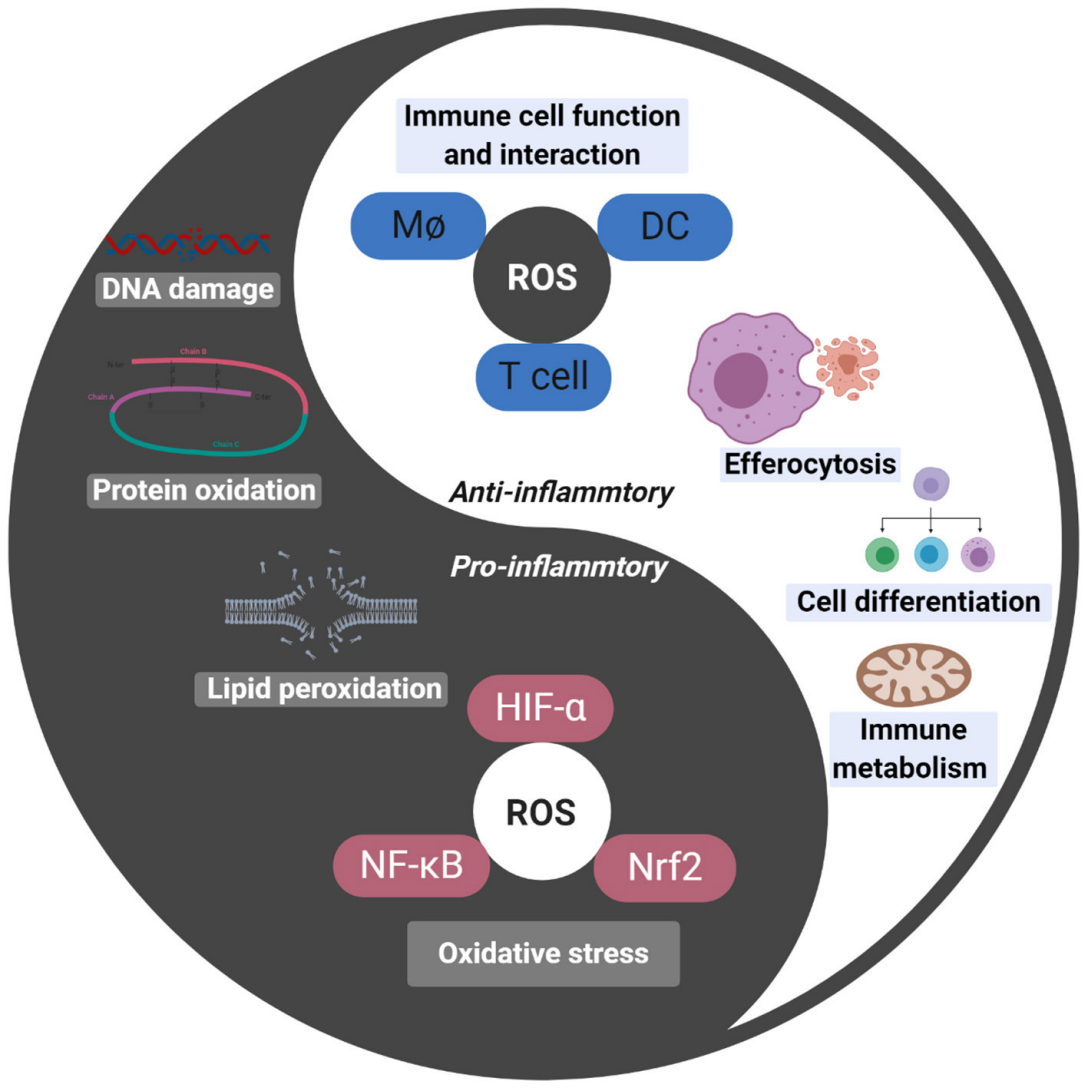
Darkness (oxidative stress) and light (signal molecule) of ROS. Oxidative stress induced by ROS lead to DNA damage, protein oxidation and lipid peroxidation, thus injuring cells. ROS also are involved in HIF-α, NF-κB, and Nrf2 mediated pro-inflammatory response. ROS act as important signal molecular in cell, which connect innate immunity and adaptive immunity, as well as participate in cell biological behaviors like metabolism, differentiation, and apoptosis.

Numerous studies have demonstrated the role of oxidative stress in the pathogenesis of autoimmune disease, varying from biomacromolecule damage to pro-inflammatory responses (3); however, antioxidant supplements may be not beneficial for primary or secondary prevention (4, 5); indeed, beta carotene, vitamin A, and vitamin E supplements may increase mortality (6). In recent years, ROS has become widely regarded as a signaling molecule, involved in many immune cell relationships and functions (Figure 1). In this review, we discuss the importance of ROS in adaptive immune responses, and the damage to immune tolerance caused by excessive ROS elimination. Further, we provide a detailed review of the roles of redox regulation in the glycolytic/PPP equilibrium in CD4^+^ T cells, focusing on rheumatoid arthritis (RA) as a model autoimmune disease.

## ROS and Immune Cells

### ROS Production and Disease

There are two main physiological sources of ROS: the NADPH oxidase complex, NOX2, and mitochondria. NOX2 is a multi-component enzyme system, composed of three cytoplasmic protein subunits (p47phox, encoded by *NCF1*; p67phox, encoded by *NCF2*; and p40phox, encoded by *NCF4*), two transmembrane protein subunits (p22phox, encoded by *CYBA* and gp91phox encoded by *CYBB*), and a small GTP-binding protein (Rac) (7, 8). In mitochondria, ROS production occurs when O_2_ receives an electron from the mitochondrial complex, which is a complex process influenced by the concentration of electron donors and O_2_, and the reactions rate constants between them (9). Chronic granulomatous disease (CGD) is an inherited disease, characterized by non-functional NOX2. Patients with CGD always suffer from recurrent life-threatening infections, due to deficient neutrophil- and macrophage-mediated innate immune responses. Interestingly, patients with CGD patients also have an increased risk of developing autoimmune diseases, resulting from their adaptive immune response disorder (8). In contrast, mitochondrial disease pathology is invariably considered to involve elevation of ROS (10); however, a study found that double mutants of alternative oxidases and severe myopathic skeletal muscle-specific *COX15* gene mutation led to decreased ROS production, and consequent impairment of PAX7/MYOD-dependent muscle regeneration. This study indicated the benefits of mitochondrial ROS (mtROS) signaling and the potential hazards arising from ROS elimination (11).

### ROS in Antigen Presentation

Mononuclear phagocytes and dendritic cell are the main professional antigen-presenting cells (APCs), in which exogenous antigens are proteolytically processed, then complexed, generally with MHC class II, or with MHC class I by a special process referred to as cross-presentation. NOX2-derived ROS in phagosomes can kill ingested pathogenic microorganisms and prevent excessive reduction of proteolysis and disulfide bond formation, by modulating the redox microenvironment, including the pH and oxidative modification of cysteine residues (12–14). In this way, the stability of effective epitopes of antigenic peptides and efficiency of their presentation are enhanced, so that APCs can better activate T cells. For example, activation of CD4^+^ T cell clones is regulated by NOX2-derived ROS through alteration of phagosome cysteine cathepsin activity, based on the immunodominant peptide epitope presented in the context of MHC Class II (14). In contrast, dendritic cells from p47phox-null mutant NOD mice (a spontaneous mouse model of autoimmune diabetes) and patients with CGD showed reduced ability to activate CD8^+^ T cells, due to antigen degradation and deficient antigenic peptide loading on MHC Class I (15). In addition, discovery of many oxidation autoantigens in APCs from individuals with autoimmune diseases indicated that ROS can change antigen structure directly, thus affecting T cell behavior (16, 17).

The other main source of ROS, MtROS may also influence the antigen presentation process in more complex ways. One study found that increased mtROS in aged murine dendritic cells (DCs) hampered the cross-presentation process, which could be restored by scavenging of ROS *in vitro*. This change is not influenced by phagocytosis function and pH (18); however, in plasmacytoid dendritic cells (pDCs), mtROS-dependent pH alkalization and antigen protection are key factors in induction of cross-presentation. This obvious difference may result from specialized toll-like receptor (TLR) activation and NOX2 independence of pDCs (19, 20). In pDCs, ROS also participates in responses to damage-associated molecular pattern (DAMP) molecules (such as mitochondrial DNA), and influences their capacity to stimulate pDCs (21). NOX2-derived ROS and mtROS may act synergistically, since one study found that, in macrophages that had already engulfed bacteria, mitochondria translocated and juxtaposed to the phagosome (22), and mtROS can be packaged by Parkin-based mitochondrial vesicles and transferred to bacteria-containing phagosomes (23).

Costimulatory molecules on the surface of APCs also influence the activation of adaptive immune cell. NOX2-derived ROS in dendritic cells endocytosing tumor cell-derived microparticles can upregulate the costimulatory molecules, CD80 and CD86, thereby activating CD8^+^ T cells. The underlying mechanism involves generation of the ROS-activated calcium channel, Mcoln2, in the lysosomal membrane, leading to Ca^2+^ release and activation of the transcription factor EB (TFEB), which can bind to the promoters of the genes encoding CD80 and CD86 (24, 25); however, in human primary monocytes infected by Epstein-Barr virus, TLR signaling activation increases ROS production. Further, ROS is an important contributor to marked up-regulation of the inhibitory costimulatory molecule, PD-L1, leading to immune escape (25); interestingly, the antioxidants, N-acetyl cysteine (NAC) and apigenin, can offset this change (26). In addition, alloantibody-FcγR I/FcγR III-dependent ROS production in macrophages is an important mediator of humoral immune damage during liver graft rejection (27). Opsonization of IgG on IFN-γ-activated macrophages led to diminished phagosomal processing of proteins in a PKC/Syk- NOX2-dependent manner, which occurs at the level of the individual phagosome (28). Altered IgG subtype distribution and the resulting increase in IFN-γ production are observed in both patients with CGD and a CGD mouse model (29), indicating a possible feedback loop involving IFN-γ, IgG, FcγRs, and NOX2.

### ROS and Macrophage Efferocytosis

Macrophages are responsible for anti-pathogen immunity and ROS is a powerful weapon in this context. Reactive oxygen species promotes both M1 and M2 macrophage polarization (30), which appears to be contradictory, as the M1 phenotype is pro-inflammatory while M2 polarization is anti-inflammatory; however, the mechanism occurs in the context of mixed M1/M2 populations present under physiological conditions, defective innate immunity, and the tendency toward autoimmunity in patients with CGD. Normally, macrophages do not require antioxidants to protect themselves from ROS-related oxidative stress, because they possess defensive measures against ROS-mediated damage, such as the Mst-Nrf2 axis (31). In contrast, the addition of anti-oxidants may disturb macrophage-mediated autoimmunity, by altering macrophage polarization and homeostasis (30).

Efferocytosis is the process by which macrophages engulf and clear billions of apoptotic cells. Impaired efferocytosis plays a vital role in inflammation in CGD patients. Peritoneal, bone marrow-derived, and alveolar macrophages from NOX2-deficient mice and primary macrophages from CGD patients showed diminished efferocytosis of apoptotic Jurkat T cells and human neutrophils both *in vivo* and *in vitro* (32, 33). The mechanism involved has several aspects: “find me” signals in the prepare phase, “eat me” signals in the implementation phase and “digest me” in the rehabilitation phase.

“Find me” signals are some soluble substances released by apoptotic cells themselves, which recruit macrophages and reshape their scavenging potential. Among these signals phingosine-1-phosphate and some metabolites (AMP, GMP, creatine, spermidine, and glycerol 3-phosphate) are reported as phagocyte gene expression modulators (34, 35). Interestingly, this characteristic of apoptotic cells seems to be changed in CGD mice. In zymosan A induced self-limited peritonitis CGD mice, researchers observed reduced macrophages/monocyte infiltration and delayed neutrophils clearance as well as diminished macrophage efferocytosis. The mechanism lays in defective respiratory burst in CGD neutrophils, thus failed to deplete local O_2_ and produce enough ROS to maintain HIF-1α protein stability that is essential to upregulate macrophage efferocytosis enhancer erythropoietin- PPARγ signals (36).

As for “eat me” signals exposed on the surface of apoptotic cells, phosphatidylserine (PS) is the strongest (37). Apoptotic neutrophils in patients with CGD are prevented from PS externalization, as this process requires the participation of NOX2-derived ROS (33, 38, 39), which is verified by treatment of normal neutrophils with NOX2 inhibitor diphenyleneiodonium (33). And peroxidized PS species (PSox) are even stronger “eat-me” signals than PS alone (40). Further, PS exposure seems to modulate macrophage program such as classical and alternative activation in M1/M2 balance, above and beyond its effect on phagocytosis (32). M2, rather M1, macrophages are the protagonists of efferocytosis; and CGD patients and NOX2-deficient mice have macrophages with an M1 phenotype that tend to promote inflammation (32, 41, 42). Finally, the difference in efferocytosis ability between M1 and M2 macrophages is primarily attributable to the central role of interleukin 4 (IL-4) signaling through peroxisome-proliferator activated receptor γ (PPARγ). *Ex vivo* treatment of macrophages from patients with CGD and NOX2-deficient mice with IL-4 or IL-13 leads to re-establishment of normal efferocytosis, as do monocytes treated with the PPARγ agonist, pioglitazone (a drug for treatment of type 2 diabetes) (32, 43). PPARγ agonist treatment can not only reverse impaired efferocytosis in CGD monocytes, but also enhances mtROS production (43, 44). Interestingly, mtROS production can promote M2 macrophage polarization in the intestine (45).

Works go on in macrophage after ingesting apoptotic cells. In contrast to function of preventing excessive antigen reduction characterized in previous section, NOX2-derived ROS in the degradation of apoptotic cells seems to be a positive correlation: efferosomes maturation (acquisition of LC3 and LAMP-1), enhanced acidic environment mediated by V-ATPases, competent proteolytic activity, and these are obviously delayed in macrophage of CGD patients. The key element of this difference lies on the nature of phagosomal protein cargo. Apoptotic neutrophils cargo contributes to activation of macrophage NOX2 in a CD11b-TLR2/TLR4-myeloid differentiation primary response 88 (MyD88)-dependent manner and the subsequent ROS production, which is significantly delayed in macrophages from NOX2-deficient mice (46). While IgG-opsonized antigen cargo activates NOX2 dependent on FcγR-PKC/Syk pathway rather than V-ATPase (13, 28, 46).

NOX2 deficiency has been identified as associated factor in autoimmune disease. For example, Ncf1 polymorphism is a stronger genetic factor of systemic lupus erythematosus (SLE) (47). It is well-known that autoantigen triggered autoantibody plays a vital role in pathology of SLE, and this process is dramatically enhanced in experimental lupus in NOX2 deficient mice (48, 49). Failure in timely clearance of apoptotic cells that is founded in NOX2 deficient lupus mice contributes to accumulation of secondary necrotic cells leading to increased secretion of inflammatory cytokines and chemokines (50).

### The Effects of ROS on T Helper 1/T Helper 2 and T Helper 17/Regulatory T Cell Balance

It is widely accepted that ROS is essential in adaptive immunity. T cell receptor (TCR) activation is accompanied by production of large amounts of ROS over a few minutes (51), which is associated with mTOR/AMPK axis-mediated metabolic reprogramming (52, 53). Reactive oxygen species is a critical link in the signaling events mediating T cell activation, proliferation, and differentiation (54, 55); however, number of studies have contradicted these findings (56). Here, we review understanding of the function of ROS in T helper 1 (Th1)/T helper 2 (Th2) cell, and T helper 17 (Th17) cell/regulatory T cell (Treg) balance, the importance of which in autoimmunity is universally acknowledged.

Th1/Th2 were the first CD4^+^ T helper cell subsets determined to contribute to autoimmune diseases (57). Characterized by IFN-γ and IL-2, Th1 cells mainly function in cellular immunity, while Th2 cells are focused on humoral immunity. The Th1/Th2 equilibrium manifests in both directions during autoimmune disease; for example, the Th1 predominance in RA (58) and Th2 advantage in SLE (59). Further the Th17/Treg equilibrium has a major role in inflammatory and autoimmune diseases (60). Interconnected developmental pathways facilitate the plasticity between Th17 and Treg phenotypes in various inflammatory (61) and oxidative (56) microenvironments.

Evidence from patients with CGD and NOX2-deficient mice includes experimental data on the “third signal” function of ROS in Th1/Th2 and Th17/Treg balance (Table 1). In NOX2-deficient mice, the T cell phenotype is skewed toward the Th1 and Th17 lineages (62, 63, 67), while macrophage-restricted restoration of ROS production improved resistance to collagen-induced arthritis in NOX2-deficient mice (68). This effect may depend on Treg induction by macrophage-derived ROS, and was confirmed in experiments using macrophages from patients with CGD (64). Further, compared with wild-type mice, Tregs from NOX2-deficient mice exert much weaker inhibition of CD4^+^ effector T cells (65), and antioxidant NAC or NOX inhibitors also induce changes in the Th1/Th2 and Th17/Treg balance (Table 2).

**Table 1:** Th1/Th2 and Th17/Treg related change in NOX2-deficient mice or CGD patients.

**Cell type**	**NOX2 mutation**	**Th1 change**	**Th2 change**	**Th17 change**	**Treg change**	**Other**
CD4^+^ T cell of C57BL/6 (62)	gp91*^*phox*^*-	IL-2 ↑	IL-4 ↓ IL-5 ↓	NA	NA	TNF ↑
Total splenocytes of C57BL/6 (63)	gp91*^*phox*^*-	IFN-γ ↑	NA	IL-17↑	NA	NA
Naïve CD4^+^ T cells of C57BL/6 (63)	gp91*^*phox*^*-	IFN-γ ↑T-bet ↑	IL-4 ↓ IL-4Rα↓ IL-5 ↓ GATA-3↓	IL-17↑	NA	STAT5Phosphorylation↓NA
PBMC from CGD patients (64)	p47*^*phox*^*-	NA	NA	NA	No change of CD4^+^CD25+ Foxp3+ cells	NA
Total spleen cell from C57BL/6 (65)	p47*^*phox*^*-	NA	NA	NA	No change of CD4^+^ Foxp3+ cells	NA
Spleen CD4 T cells of OT-II mouse (66)	p47*^*phox*^*-	IL-2 ↓IFN-γ↓CD4^+^T-bet+ cell↓		IL-17A↓	IL-10↓	TNF-α↓TGF-β↓IL-5↓Il-12p70↓

*Frequencies of Th1, Th2, Th17, Treg cells, or levels of related cytokines are compared between immune cells from NOX2 mutation mice/CGD patients and wild type mice/healthy people. ↑: levels of cytokines or frequencies of cells are higher than wild type mice/healthy people. ↓: levels of cytokines or frequencies of cells are lower than wild type mice/healthy people*.

**Table 2:** Th1/Th2 and Th17/Treg related change in different treatments.

**Cell type**	**Treatment**	**Th1 change**	**Th2 change**	**Th17 change**	**Treg change**	**Other**
CD4^+^ T cell from BALB/c mice (63)	10 mM NAC (ROS scavenger)	IFN-γ ↑	IL-4 ↓ IL-5 ↓	NA	NA	STAT5Phosphorylation↓
Human CD4+CD25– T cells (64)	Primed with macrophage from p47*^*phox*^*-CGD patients	IFN-γ ↑	NA	IL-17↑	CD4+CD25+Foxp3+ cell ↓	NA
CD4+ CD45RO– T-cells under Th1- and Th17-skewing conditions (69)	Tempol (ROS scavenger)	IFN-γ+ cells ↑	NA	IL-17+ cells ↑	NA	NA

*Thayer et al. (70) immune cells whether receiving ROS clearing treatment, or primed with macrophage from CGD patients and healthy people. ↑: levels of cytokines or frequencies of cells are higher than control group. ↓: levels of cytokines or frequencies of cells are lower than control group*.

However, it is noteworthy that NOX2 deficiency in combination with transgenic mice shows different change of T cell subsets. For example, NOX2 deficiency in OT-II mice (a transgenic mouse model with antigen-specificity for chicken ovalbumin 323-339 in CD4^+^ T cell) lead to both decreased Th1 and Th17 lineages in contrast with NOX2-deficient mice (66). Further, NOX2 deficiency in NOD mice serves as protector reflected in significant reduction and delay in autoimmune diabetes development (67, 70, 71). And macrophage, CD4^+^ T cell, CD8^+^ T cell are involved in the protection (Table 3). Weakened Th1 lineage proficiency resulted from absence of Th1 transcription factors (T-bet, STAT4, and STAT1α) and Th17 proneness by STAT3 activation are observed in NOX2-deficient NOD mice (67). Followed research revealed that the protection afforded by NOX2 deficiency is rely on ROS lack in macrophages and DCs leading to reduced CD4^+^ T-cell autoreactivity [one possible mechanism is reduced MHC-II complex by macrophage (72)] and CD8^+^ T cell effector function [one possible mechanism is defective cross presentation by DC (15)], rather than isletβ-cell and neutrophils (70). Surprisingly, NOX2 deficiency in NOD.BDC-2.5 mice (a TCR transgenic mice whose TCR specifically recognizes islet antigen) that was meant to prevent from autoimmune diabetes has instead resulted in spontaneous type 1 diabetes. In followed adoptive transfer experiment, CD4^+^ T cells of NOX2-deficent NOD.BDC-2.5 mice were more diabetogenic upon adoptive transfer into NOD due to less suppressive Tregs (73). These studies implied the complication of ROS in immune cell response to heterogeneous microenvironment.

**Table 3:** Th1/Th2 and Th17/Treg related change in NOX2-deficient (*NCF1* mutation) NOD mice.

**Cell type and mice**	**Type 1 diabetes**	**Macrophage related change**	**CD4/CD8+ T cell related change**	**Other**
CD4^+^ T cells of NOD mice (67)	Remission	NA	IFN-γ ↓, IL-2 ↓, T-bet ↓ IL-4 ↓, IL-17↑, IL-10↑ CD4+/IL-17A+ cell↑ No change of CD4+CD25+ Foxp3+ cells	TNF-α↓ TGF-β↑
Spleen CD8^+^ T cells (70) of NOD mice	Remission	NA	CD8+/IFN-γ+ cells↓ CD8+/GzmB+ cells↓	NA
BM-Mφs and CD4^+^ T cell of NOD mice (72)	Remission	MHC-II↑, TNF-α↓, IFN-β↓, TLR3↓, NF-κB↓	No change of CD4+ Foxp3+ cells	NA
Islets and BM-Mφs with M1/M2 Polarization of NOD mice; (71)	Remission	M1 Marker↓ (cxcl10, ccl5, iNOS, TNF-α, IFN-γ,STAT1 )M2 Marker↑ (ccl17, Arg1, Retnla, CD206, STAT6)	NA	NA
Splenocytes of NOD.BDC-2.5 mice (73)	Exacerbation	NA	IFN-γ↑, IL-17↑ IL-2↑, IL-12Rβ2↑ Activation Markers↑ (CD25, CD44, CD69)	TNF-α↑IL-1β↑

*Frequencies of Th1, Th2, Th17, Treg cells, or levels of related cytokines are compared between immune cells from NOX2 mutation NOD mice and NOD mice. ↑: levels of cytokines or frequencies of cells are higher than NOD mice. ↓: levels of cytokines or frequencies of cells are lower than NOD mice. BM-Mϕs, Bone marrow macrophages*.

A key factor influencing Th1/Th2 differentiation from Th0 cells is the cytokine microenvironment, where APC represent a cytokine source. If glutathione (GSH; a major cellular antioxidant) is depleted in APCs over a short time period, production of Th1-associated cytokines will be inhibited and Th2-associated cytokine generation favored (74). Similarly, low doses of H_2_O_2_ can prevent activated Th1 clones from producing INF-γ and potentiate IL-4 secretion by activated Th2 clones (75). T cell activation-triggered IL-2 and IL-4 expression is partly dependent on ROS generation and follows oxidative signaling via mitochondrial respiration chain complex I. Inhibition of complex I function will decrease mtROS generation, thus blocking activation-induced secretion of IL-4 in CD4^+^ T cells from patients with atopic dermatitis, a disease characterized by elevated IL-4 and IgE. Prolonged ciprofloxacin treatment has the same effect on CD4^+^ T cells as the complex I inhibitor, rotenone, which may explain the immune regulation function of ciprofloxacin (76). The transcription factor, Bach2, plays a vital role in shaping the balance between CD4^+^ T cell subsets (77). TCR signaling induced by ROS specifically limits the degradation of the SUMO-specific protease, SENP3, leading to its rapid accumulation in Tregs. SENP3 promotes Bach2 deSUMOylation and prevents its nuclear export, which inhibits the expression of IFN-g, IL-17, and other effector cytokines, and maintains Treg-specific gene signatures (78). Reactive oxygen species promotes PAC1/DUSP2 expression by activating the transcription factor, EGR1, while PAC1 suppress the STAT3 signaling crucial for Th17 lineage differentiation (79, 80). In addition, we have described above that the post-translational oxidative modification is the main change in aging (2). Protein oxidative modification state is regulated by balance between ROS and methionine sulfoxide reductase (Mrs). Mrsb1 deficiency in DC delays its maturation and decreases DC-induced Th1 differentiation, and is associated with defective differentiation of follicular helper T cell cells *in vivo* (81).

**Figure 2:**
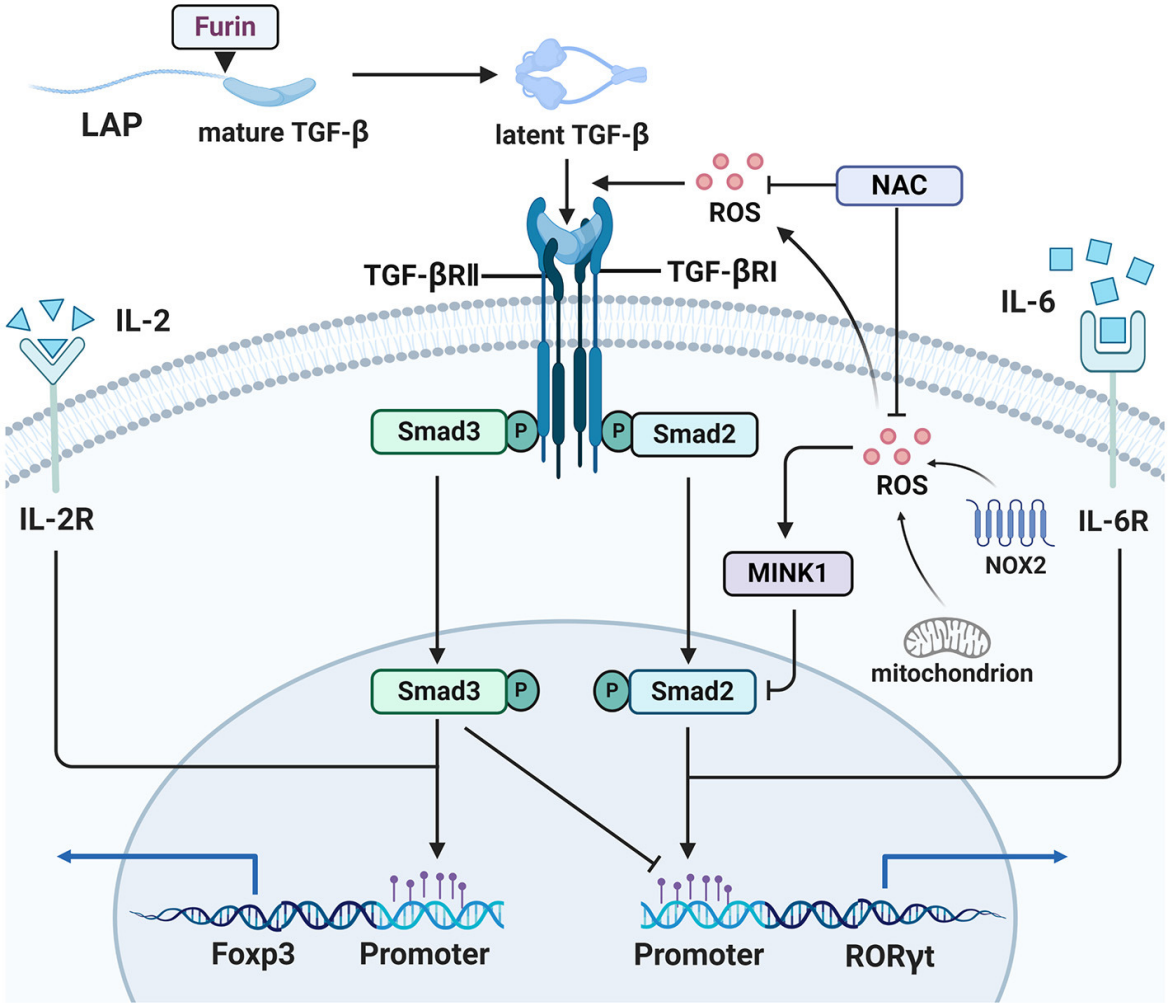
ROS participate in Th17/Treg balance. ROS are involved in TGF-β mediated Th17/Treg balance. Latent TGF-β formed after LAP in the TGF-β precursor was cut by Furin, and then ROS activated TGF-β from its latent form. NOX2 or mitochondrion derived ROS activated MINK1 that inhibits the phosphorylation of Smad2 in T324 residue, so that expression of Th17-associted genes is blocked, and ROS scavenger NAC will reverse this function. ROS also participate in expression of Treg-associated genes.

Transforming growth factor β (TGF-β) is a cytokine with broad regulatory functions in T cell development and differentiation. The complexity of TGF-β-related signaling is partly reflected in its contradictory functions and mechanisms in Th17/Treg differentiation. TGF-β is normally synthesized as a precursor, whose C-terminal portion is referred to as latency-associated peptide (LAP). LAP is cleaved by Furin, to generate latent TGF-β, which can occur both intracellularly and extracellularly (82). Latent TGF-β can be activated by various molecules, including integrin αvβ8 and ROS in T cells (83, 84). Human CD4^+^ CD25^−^ naïve T cells can be induced to express Foxp3 by stimulation with anti-TCR and anti-CD28 antibodies plus ROS (85). This effect relies on production of latent TGF-β by TCR and CD28 engagement, as well as subsequent activation of latent TGF-β by ROS on TCR stimulation (84). Promotion of Th17 generation by high glucose and Treg generation by D-mannose are dependent on addition of exogenous latent LAP-TGF-β, rather than active TGF-β, indicating that activation related signals contribute to this effect, but not active TGF-β itself (86, 87) (Figure 2). Why active TGF-β does not function in this context and how the “activation-related signals” promote TGF-β signal transduction remain unclear; we speculate that intercellular communication, controlled by receptor-mediated TGF-β activation, may be a contributing factor, while “activation-related signals” may trigger some change that influences TGF-β signal transduction. Reactive oxygen species are key intermediary molecules for activation of latent TGF-β, and NAC abolishes the induction of Th17 cells and Tregs (86, 87). Reactive oxygen species not only acts as an upstream molecule to activate latent TGF-β, but also participates in TGF-β-Smad signaling. Misshapen (Msn)/NIK-related kinase 1 (MINK1) is a serine-threonine kinase that can induce Th17 differentiation by directly phosphorylating the T324 site at the α1 helix region of the Smad2 protein (88). Reactive oxygen species are involved in activation of MINK1 (89), and NAC treatment profoundly reduced MINK1 activity and increased the frequency of IL-17A^+^ cells, which did not occur in MINK1-deficient T cells. The processes described above are shown in Figure 2. Further, in Th17 cells differentiated from MOG35–55-immunized CD4^+^ T cells, NAC treatment resulted in more severe experimental autoimmune encephalomyelitis (EAE) disease after transfer into Rag1^−/−^ mice (88), while knockout of GSH in T cells led to EAE resistance in mice. IsoalloLCA is a bile acid metabolite, which can enhance mtROS production in CD4^+^ T cells. Elevated mtROS increased H3K27 acetylation at the Foxp3 promoter region in a TGF-β-Smad3 signal dependent manner, thereby promoting Treg differentiation (90). Moreover, ROS also contributes to suppression of CD4^+^ effector T cells mediated by Tregs, which is partly dependent on TGF-β and can be blocked by thiol-containing antioxidants (65).

Abnormal redox related Th subsets change has been identified in autoimmune diseases, such as RA. Naïve CD4^+^ T cells from RA patients possess a distinct carbohydrate metabolic signature, which is manifested as an excessive shunt of glucose into the PPP, resulting in high levels of reduction mediated by GSH and NADPH, and exhaustion of ROS (69, 91, 92). Low levels of ROS lead to ataxia telangiectasia mutated (ATM) deficiency, causing rapid T cell proliferation and differentiation toward Th1 and Th17 lineages (69, 91); however, in a study of inflammasomes in RA, naïve CD4^+^T cells from RA patients produced more ROS compared with those from healthy controls (93); this contradictory result may be attributable to measurement being conducted as early as 8 h after anti-CD3/CD28 stimulation, which is much earlier than the 3–6 day time points used in two other studies (69, 92). In those reports, treatment of CD4^+^ T cells from RA patients with the pro-oxidant, plumbagin, reduced the frequencies of Th1 and Th17 cells, as well as decreasing production of the inflammatory cytokines, TNF-α and IL-6. The antioxidant, NAC, completely reversed the regulatory effects of plumbagin (94). These studies demonstrate the potential pro-inflammatory function of ROS exhaustion in RA CD4^+^ T cells, and anti-inflammatory benefits of supplementation with oxidants.

Based on the evidence reviewed above, it remains unclear how strong the effect of ROS is in autoimmunity-related Th1/Th2 and Th17/Treg balance. Regarding patients with CGD, it is difficult to determine a clear role of CD4^+^ T cells in CGD-related autoimmunity risk. For example, one study reported that only gp91phox-deficient CGD is associated with diminished Tregs (95); however, a review demonstrated that oxidative stress leads to T-cell dysfunction in SLE by altering Th cell lineages and gene transcription (96).

### ROS and Inflammatory Metabolism: Kyn-IDO1

Indoleamine 2,3-dioxygenase (IDO) has maintained a central role in tryptophan metabolism over hundreds of millions of years of evolution (97, 98). It is well-established that IDO has suppression and feedback roles in immune regulation (99), and clinical trials of IDO inhibitors for treatment of cancers were very successful. The normal dioxygenase activity of IDO is post-translationally activated by biological reduction of Fe^3+^ to Fe^2+^ in heme. Of these reducing agents, ROS, and particularly superoxide anion (O2?), are most widely studied in mice and rabbit (100, 101). Specifically, hyperbaric oxygen can increase kynurenine concentration in rat brains by 60% compared with air (102). In murine atopic dermatitis and psoriasis lesions, hyperbaric oxygen therapy can elevate ROS levels to attenuate disease, which may be mediated by enhanced IDO expression and Treg function (103, 104). Precisely because of the immunosuppressive effect of IDO and its ROS dependency, defective IDO activation and tryptophan metabolism were once considered to be important factors contributing to hyper inflammatory responses in murine CGD (101, 105). Interestingly, long-term evolution appears to have freed human IDO activity from ROS restriction. The IDO metabolic activity of leukocytes and monocyte-derived dendritic cells is fully maintained and intact in human patients with CGD (106–108), indicating that ROS is not indispensable for human IDO activity. Cytochrome b5, rather than ROS, has a major role in IDO reduction in human cells (100, 109); however, it is not clear whether the entire tryptophan metabolism process is independent of ROS in humans. LPS-induced ROS promotes DC maturation mediated by IDO and NF-κB activation, leading to expansion of CD4^+^ CD25^high^ Tregs (110). A tryptophan catabolite of IDO, L-kynurenine (Kyn), can induce apoptosis of NK cells in a ROS-dependent manner. This pro-apoptotic effect can be entirely prevented by the antioxidant, NAC (111). In turn, Kyn also can elevate ROS levels in activated T cells and promote their proliferation, which relies on inhibition of sepiapterin reductase, the terminal enzyme in the *de novo* tetrahydrobiopterin synthesis pathway (112). Further, IDO1 can even produce single molecular oxygen in the presence of hydrogen peroxide. The generated ^1^O_2_ oxidizes L-tryptophan to a tricyclic hydroperoxide, thus regulating vascular tone and blood pressure under inflammatory conditions (112).

## ROS and Rheumatoid Arthritis

Due to the physiological function of ROS, high levels of reducing equivalents and excessive ROS scavenging may lead to damage of the opposite type to oxidative stress, sometimes referred to as reductive (113) or antioxidative (114) stress.

Redox regulation treatments should be disease-specific as the different redox characteristics among diseases. For example, unlike the central role of oxidative stress in lupus pathogenesis, CD4^+^ T cells of RA patients experienced reductive stress (115). In addition, variations in redox state exist among types of lesions and cells in RA. In the next section, we discuss the metabolic origin of these variations and their influences on pathology.

### ROS in RA: Every Coin Has Two Sides

RA pathogenesis is not well-understood, but can be summarized by loss of peripheral immune tolerance to autoantigens, followed by excessive activation of T and B cells, leading to increased levels of cytokines and autoantibodies (rheumatoid factor, anti-cyclic citrullinated peptide antibodies, etc.). The homeostasis between pro- and anti-inflammatory states is destroyed, eventually leading to damage of multiple joints and other organs throughout the body.

Numerous studies have confirmed the vital role of ROS-related oxidative stress in joint pathology, including in angiogenesis, synovial proliferation, and inflammatory infiltration (116, 117). Further, functional genetic analysis showed that the rs201802880 polymorphism in the *NCF1* coding region is associated with genetic susceptibility to RA (47). Patients with CGD also have increased susceptibility to RA (47, 118). Moreover, a study of NOX2-deficient mice demonstrated that the absence of ROS prevents resistance to autoimmune arthritis. A collagen-induced arthritis model generated by Ncf1 mutation in mice has more severe symptoms, higher anti-CII IgG levels, and stronger Th1 responses than wild-type mice, which can be reversed by restoration of functional Ncf1 solely in macrophages. Interestingly, this research also found that T cells from Ncf1-mutated mice responded more vigorously to APCs (68). Mice with mutated mouse collagen (MMC) have higher resistance to arthritis mediated by a mutated immunodominant epitope in collagen type II that binds to the MHC class II molecule. When these MMC mice are bred with NOX2-deficient mice, their immune tolerance to arthritis disappears, and they exhibit enhanced autoimmune T cell responses and higher anti-CII IgG levels (119).

Poly-N-isopropylacrylamide (PNiPAAm)-based polymers are new synthetic substances that can serve as adjuvants in inducing experimental arthritis. Mixture of these new “adjuvants” with natural CII triggers more severe arthritis and stronger autoantibody responses in Ncf1-mutated mice, in which macrophage ROS also plays a central role (120). DC function is also influenced by redox state. *Mycobacterium tuberculosis*-activated DC with ROS exhaustion (through incubation with SOD and catalase, or derived from NOX2-deficient mice) produced more IL-1β, TNF-α, TGF-β, and IL-6. NOX2 mutation breaks the resistance to arthritis of wild-type C57BL/6 mice, and this is CII specific, as Freund's complete adjuvant alone cannot induce arthritis. In addition, when DC from NOX2-deficient mice serve as APC, T cells produce more IL-17 after activation by toxic shock syndrome toxin-1 (TSST-1) (121); however, research into SKG mice (a spontaneous arthritis animal model caused by ZAP70 mutation) indicates that Ncf1 mutation-related ROS deficiency does not exhibit further alteration of T-cell activation or differentiation profile because of ZAP70 mutation, while transgenic restoration of functional Ncf1 in macrophages modified the arthritis in Ncf1-mutated SKG mice to the state observed in ROS-sufficient SKG mice. This research indicates that innate, but not adaptive, inflammation contributes to more severe arthritis related to ROS deficiency (122). Therapeutic strategy that increasing NOX2-derived ROS has been tested, in which phytol, an oxidative burst-inducing substance, ameliorated pristane induced rat arthritis in T cell and IFN-βdependent pathway (123, 124). Other free radicals, namely reactive nitrogen species, also contribute to arthritis pathogenesis in Ncf1-mutated mice, and may counteract the effects of ROS. Treatment with the NOS inhibitor, L-NAME, in the priming, rather than the effector, phase prevents Ncf1-mutated mice from developing CII-induced arthritis (125).

### The Reductive State in RA Naïve CD4^+^ T Cells

Subsets of CD4^+^ T cells, including Th17 cells and Tregs, are recognized as important targets for the treatment of autoimmune disease; however, few studies have concentrated on the role of naïve CD4^+^ T cells. After recognizing the MHC complex, naïve CD4^+^ T cells initiate rapid clonal expansion, with a consequent explosive increase in energy and biosynthesis demands. The energy demands are dependent on transformation from oxidative phosphorylation to glycolysis, referred to as metabolic reprogramming (52). This may appear to be a retrogression, where an efficient method of ATP generation is abandoned in favor of a wasteful method; however, glycolysis actually is the more logical choice for cells undergoing rapid proliferation. Oxidative phosphorylation has a much higher proteome costs than glycolysis, because of the prerequisite complicated mitochondrial infrastructure, that requires huge energy expenditure (126). The enhanced PPP and glutamine decomposition meet the NADPH and biosynthetic precursor needs during T cell growth and proliferation (127), and both participate in GSH generation. The balance of energy generation and biosynthesis is also very important. In contrast, deficiency of any of these processes will destroy the basic support for normal physiological activities after T cell activation. In contrast, excessive glycolysis is associated with pro-inflammatory T cell subsets (128) and excessive biosynthesis indicates a worse outcome (i.e., a tumor). In normal naïve CD4^+^ T cells, glycolysis activation occurs in response to upregulation of the glucose transporter, GLUT, which increases glucose uptake (129), and activities of several rate-limiting enzymes, including 6-phosphofructo-1-kinase (PFK-1) (130). PFK-1 activity is mainly dependent on allosteric activation by fructose-2,6-bisphosphate (F2,6P2), and production of F2,6P2 is primarily controlled by four fructose-2,6-bisphosphatase (PFKFB) isoenzymes (131), among which PFKFB3 is the strongest. During the activation of CD3^+^ T cells, PFKFB3 expression is increased in response to engagement of the TCR and the co-stimulatory receptor, CD28 (132). Two studies found that, compared with those from healthy people, naïve CD4^+^ T cells from patients with RA failed to upregulate PFKFB3 expression during the activation process, leading to reduced glycolytic flux and diminished ATP generation. As a rate-limiting enzyme of the PPP, glucose-6-phosphate dehydrogenase (G6PD) initiates the PPP by dehydrogenation of glucose 6-phosphate. Unlike PFKFB3, these naïve CD4^+^ T cells successfully upregulated G6PD expression, which controls the fate of residual glucose, namely, influx to the PPP. Hence, there is an imbalance of energy generation and biosynthesis in naïve CD4^+^ T cells from patients with RA, resulting in accumulation of GSH and NADPH, reduced ATP generation, and ROS exhaustion (Figure 2) (60, 79).

### Altered Metabolism Influences T Cell Differentiation and Proliferation

Glutaminolysis is a basic and widespread metabolic process that links oxidative phosphorylation (OXPHOS), biosynthesis, and redox regulation. The main branch point hinges on glutamate, the first product of glutamine decomposition, which can serve as material for *de novo* synthesis of GSH to regulate oxidation, or transform into α-KG and enter the TCA cycle to generate ATP, mtROS, and biosynthetic precursors. Hence, the different destinies of glutamate generate counteracting metabolites (GSH and ROS), facilitating precise coordination of metabolic flux by altering enzyme activity (133). Activation of primary T cells requires rapid glutamine uptake mediated by the amino acid transporter, ASCT2 (127). Glutaminolysis inhibition of CD4^+^ T cells has an anti-inflammatory function in autoimmunity, promotes high levels of Foxp3 expression (134) and decreases Th17 differentiation in SLE and EAE (135, 136). In RA, fibroblast-like synoviocytes (FLS) express increased levels of glutaminase 1, and inhibition of glutaminase 1 reduces RA-FLS proliferation (137); however, there has been no study of the glutaminolysis phenotype of RA naïve CD4^+^ T cells. Given the dysregulated redox state in RA T cells, whether or not glutaminolysis contributes to this phenomenon warrants discussion and study.

Other than sharing the same substrates, connections between PPP and glycolysis also include their interactions in metabolic signaling. Although excessive PPP and accumulated GSH lead to reductive stress in RA primary CD4^+^ T cells, T cells lacking GSH also appear to be incapable of initiating metabolic reprogramming, because of impaired Myc expression, NFAT activation, and mTOR activation. Higher ROS levels appear to be the protagonist, as ROS scavengers reverse the influence of lack of GSH (138). Briefly, both high levels of ROS and exhaustion of ROS harm normal transfer into glycolysis during metabolic reprogramming following T cell activation; recalling the old Chinese idiom, “Beyond is as wrong as falling short.”

Similar with the higher risk of fetal deformity occurring during the first 3 months of pregnancy, naïve CD4^+^ T cell may represent a stage at which pathogenic factors can readily influence T cells, with pathological changes at this stage having profound and lasting influences. Reactive oxygen species exhaustion disturbs normal DNA repair capabilities in naïve CD4^+^ T cells from patients with RA, and ATM insufficiency is a key factor influencing this phenomenon (69, 139). The resulting accumulation of DNA damage, ATP deficiency because of reduced glycolysis, and impaired autophagy induction (92) mean that naïve CD4^+^ T cells are more sensitive to apoptosis, leading to an abnormal loss of T cells. Since patients generally present with RA in middle-age, it is T cell auto proliferation, rather than newborn T cells, that maintain homeostasis of the T cell compartment. Remaining T cells are confronted with replicative stress, under the influence of lymphopenia, excessive biosynthesis, and pro-inflammatory cytokines. Consequently, the T cell immune aging process begins (140, 141). In addition, this imbalance supports pro-inflammatory functions, such as Th17 lineage expansion and enhanced synovial invasiveness (69, 142).

### Division of Glycolysis Between Naïve CD4^+^ T Cell and Synovium in RA: Hypoxia, Lactic Acid, and ROS

**Figure 3:**
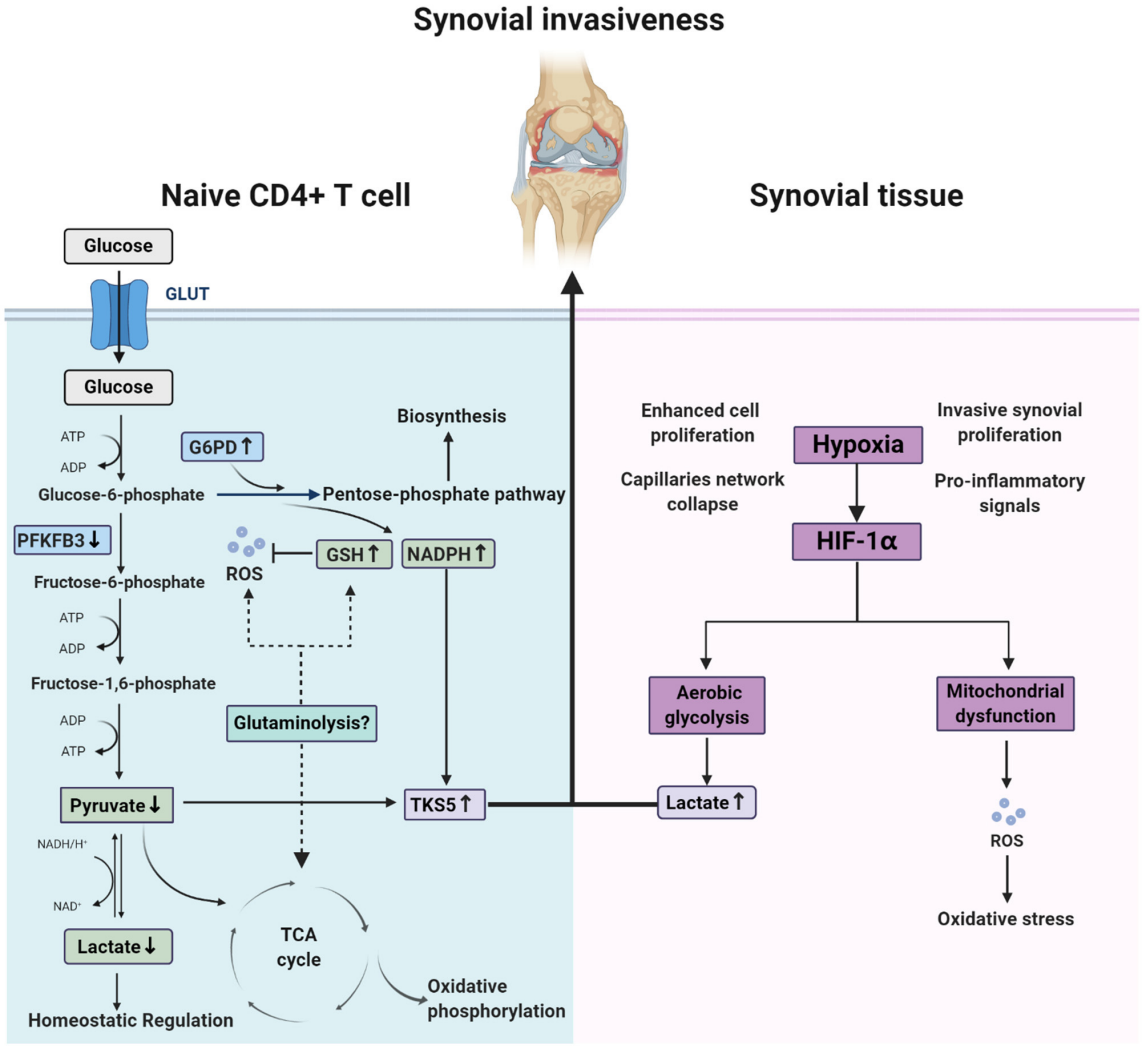
Different redox state between naïve CD4^+^ T cell and synovium in rheumatoid arthritis. In naïve CD4^+^ T cell of rheumatoid arthritis patients, glucose shunting into pentose phosphate pathway due to the change of metabolic key enzymes (PFKFB3 and G6PD) activity leads to a special microenvironment characterized by low ATP, high reducing equivalences (GSH and NADPH) and exhausted ROS. Whether glutaminolysis is associated with this remains to unclear. In contrast, synovium of patients experienced oxidative stress and abnormal aerobic glycolysis that result from hypoxia in the joint. These two split situation destruct joints together by the integration of up-regulated TKS5 in naïve CD4^+^ T cell and high lactates in synovium.

Hypoxia has been identified as a constant feature of RA synovial tissue, which occurs in the pre-arthritic phase because of increased cell proliferation, capillary network collapse, and maintenance of the inflammatory phase, due to invasive synovial proliferation, dysregulated architecture of the microvasculature, and pro-inflammatory signals, such as HIF-1α and JAK–STAT signaling (143). In energy metabolism, hypoxia is always accompanied by aerobic glycolysis and mitochondrial dysfunction, leading to accumulation of lactic acid and ROS (9). Indeed, accumulated lactic acid supports pro-inflammatory T cells to remain at the double-positive stage and produce more IL-17 (144, 145), while ROS causes oxidation with broad impacts (116, 117); however, as discussed above, in CD4^+^ T cells at the preliminary stages of RA, glycolysis is decreased and ROS is exhausted, resulting in a reductive metabolic microenvironment (low pyruvate and high NADPH) and triggering aberrant lipogenesis. This induces up-regulation of the podosome scaffolding protein, TKS5, and formation of cell membrane structures beneficial to T cell synovial invasion (142). Interestingly, this division of glycolysis between RA T cells and the synovium may combine to induce joint destruction: pro-inflammatory T cells invade synovial tissue quickly and easily, while departure is more difficult (Figure 3). In addition, lactate, which was once considered a metabolic waste product, is now thought to act as a homeostatic regulatory substance capable of counteracting the inflammatory responses caused by HIF1α and glycolysis metabolites, such as macrophage polarization, tumor immunity, and antiviral responses (146), and the newly discovered histone lysine lactate modification, lactylation, may be an important mechanism underlying these processes (147). Therefore, the decreased lactate levels in RA naïve CD4^+^ T cells, due to deficient glycolysis, may also contribute to the immune pathology of RA.

## Progressive Opinion in Redox Modulation

Studies indicate that oxidatively modified lipids, proteins, and nucleic acids, may be typical of atherosclerosis. Oxidation of low-density lipoprotein has been clearly identified as an important initial event for the onset of atherosclerosis (148). Further, regarding oxidative stress in immune-related disease, oxidatively modified autoantigens are a major topic of interest, because of their induction of loss of immune tolerance. Nevertheless, cardiovascular patients do not benefit from antioxidant supplements (5), and their effects in autoimmune diseases, such as RA, are highly contentious (149–151).

Several points may explain the failure of “one-size-fits-all” antioxidant supplements in human studies. First, the reactivity of antioxidants is dependent on the oxidants they encounter, and rate constants are highest in specific pairs: α-tocopherol and peroxyl radical, glutathione and peroxynitrous acid, ascorbic acid and carbonate anion radical, glutathione and hypochlorous acid, and β-carotene paired with singlet oxygen (152). In view of different types of oxidative modification of biological macromolecules in various degenerative and aging related diseases, as well as selectively or indiscriminately produced oxidation products, application of bulk antioxidants are expected to be more precise and targeted. Second, bioavailability in target organs is a key factor. Take ischemic stroke for example, edaravone works by eliminating free radicals and suppressing oxidative stress. However, low bioavailability and inefficient penetration across the blood-brain barrier limits the curative. Treatments based on drug nano-systems loading with edaravone possess better scavenging efficiency of free radicals (153, 154). Third, different roles of antioxidants between target organ and the others may be an important reason for side effects. In view of ROS as the key beneficial messenger in the barrier ecosystem, oral administration of antioxidants which is the main application way by people, may starts its disturbance on the body upon first barrier—gastrointestinal tract (155).

Inspired by these findings in exogenous antioxidants, new strategies may combat oxidative stress shifts by promoting innate redox modulation systems; For example, by increasing the level of endogenous NADPH (156). Another meaningful progress is the use of the SOD mimics. Superoxide dismutase 2 (MnSOD) is a part of innate redox modulation systems, which plays an important role in regulating the ROS level in mitochondria. Based on therapeutic potential of MnSOD in human diseases, SOD mimics have been developed and are currently in several clinical trials (157). Interestingly, due to the redox potential falling in between the potentials for the oxidation and reduction of O_2_•-, these MnSOD mimics are capable of role transformation from reductants to oxidants decided by chemical properties of the reaction and the cellular environment (157). Except for drug therapy, there are some special treatments promoting redox balance and deserve attention. A recent study found static magnetic and electric fields rapidly ameliorate insulin resistance and glucose intolerance in type 2 diabetes dependent on mitROS induction, and SOD2 application fully abolishes these positive effects (158). Further, exercise especially acute exercise is widely believed a process producing large numbers of ROS which causes skeletal muscle damage, fatigue and impair recovery. As a result, antioxidants supplement has become common practice. However, several reviews have well-characterized that increase of free radicals explain the health promotion effect of exercise, and antioxidants supplement reduce the positive effects of exercise (159–161). These discoveries imply the potential bidirectional therapy, which fits the point we mentioned in this review: enhancement of ROS may sometimes be required in diseases.

## Conclusion

Unlike their past reputation as harmful factors, a focus on ROS as important signaling molecule has developed in recent years. A typical example of this change in research direction is illustrated by the fact that tissue-specific redox modification of proteins has replaced biomacromolecule damage as the main agent involved in the process of aging. Failures of clinical trials into antioxidant supplements led scientists to deeply consider the problems of whether and how antioxidation strategies should be implemented.

This review has discussed the important roles of ROS in various autoimmune functions. For example, ROS influences interactions between innate and adaptive immunity by controlling the antigen presentation and apoptotic cell clearance. Evidence from NOX2-deficient mice and patients with CGD support the functions of ROS in regulating Th1/Th2 and Th17/Treg balance. Immunometabolism is an important process to which ROS contributes. Tryptophan metabolism deficiency contributes to the stronger, harmful inflammatory response in CGD mice. Regarding autoimmune diseases, such as RA, alterations in glucose metabolism-related redox imbalance have broad impacts. Due to glucose shunting into the PPP from glycolysis, naïve CD4^+^ T cells from RA patients are a good cellular level model system to explore T cell immune responses in a naturally ROS deficient environment. This special change in metabolism and redox balance leads to DNA repair deficiency, susceptibility to apoptosis, and differentiation into inflammatory subsets of RA naïve CD4^+^T cells. In addition, although the metabolism and redox state in RA synovial tissue are completely contrary to that of naïve CD4^+^T cells, we speculate that they act in combination to mediate joint destruction; however, the origin of this specific type of metabolic reprogramming remains unclear, and whether ROS contributes to triggering this process also awaits further investigation.

Overall, ROS clearance may be beneficial in specific situations, but harmful in others. Under no circumstances should we regard antioxidant supplements as completely safe treatments, particularly for immune disease. Moreover, antioxidant supplements are not equivalent to ROS clearance.

## Author Contributions

WL: writing, original draft, and figures. PS: review and editing. YS: resources. YH: resources. ST: conceptualization. All authors contributed to the article and approved the submitted version.

## Conflict of Interest

The authors declare that the research was conducted in the absence of any commercial or financial relationships that could be construed as a potential conflict of interest.
